# Olive oil's bitter principle reverses acquired autoresistance to trastuzumab (Herceptin™) in HER2-overexpressing breast cancer cells

**DOI:** 10.1186/1471-2407-7-80

**Published:** 2007-05-09

**Authors:** Javier A Menendez, Alejandro Vazquez-Martin, Ramon Colomer, Joan Brunet, Alegria Carrasco-Pancorbo, Rocio Garcia-Villalba, Alberto Fernandez-Gutierrez, Antonio Segura-Carretero

**Affiliations:** 1 Catalan Institute of Oncology (ICO)-Health Services Division of Catalonia, Spain; 2 Metabolism and Cancer Laboratory, Girona Biomedical Research Institute (IdIBGi), Spain; 3 Medical Oncology, Dr. Josep Trueta University Hospital of Girona, Girona, Catalonia, Spain; 4 M.D. Anderson International Madrid, Madrid, Spain; 5 Department of Analytical Chemistry, Faculty of Sciences, University of Granada, Granada, Spain

## Abstract

**Background:**

A low incidence of breast cancer in the Mediterranean basin suggests that a high consumption of Extra Virgin Olive Oil (EVOO) might confer this benefit. While the anti-HER2 oncogene effects of the main ω-9 fatty acid present in EVOO triacylglycerols (*i.e*., oleic acid) have been recently described, the anti-breast cancer activities of EVOO non-glyceridic constituents -which consist of at least 30 phenolic compounds-, remained to be evaluated.

**Methods:**

Semi-preparative HPLC was used to isolate EVOO polyphenols (*i.e*., tyrosol, hydroxytyrosol, oleuropein). Both the anti-proliferative and the pro-apoptotic effects of EVOO phenolics were evaluated by using MTT-based quantification of metabolically viable cells and ELISA-based detection of histone-associated DNA fragments, respectively. The nature of the interaction between oleuropein aglycone and the anti-HER2 monoclonal antibody trastuzumab (Herceptin™) was mathematically evaluated by the dose-oriented isobologram technique. HER2-specific ELISAs were employed to quantitatively assess both the basal cleavage of the HER2 extracellular domain (ECD) and the expression level of total HER2. The activation status of HER2 was evaluated by immunoblotting procedures using a monoclonal antibody specifically recognizing the tyrosine phosphorylated (Phosphor-Tyr1248) form of HER2.

**Results:**

Among EVOO polyphenols tested, oleuropein aglycone was the most potent EVOO phenolic in decreasing breast cancer cell viability. HER2 gene-amplified SKBR3 cells were ~5-times more sensitive to oleuropein aglycone than HER2-negative MCF-7 cells. Retroviral infection of the HER2 oncogene in MCF-7 cells resulted in a "SKBR3-assimilated" phenotype of hypersensitivity to oleuropein aglycone. An up to 50-fold increase in the efficacy of trastuzumab occurred in the presence of oleuropein aglycone. A preclinical model of acquired autoresistance to trastuzumab (SKBR3/Tzb100 cells) completely recovered trastuzumab sensitivity (> 1,000-fold sensitization) when co-cultured in the presence of oleuropein aglycone. Indeed, the nature of the interaction between oleuropein aglycone and trastuzumab was found to be strongly synergistic in Tzb-resistant SKBR3/Tzb100 cells. Mechanistically, oleuropein aglycone treatment significantly reduced HER2 ECD cleavage and subsequent HER2 auto-phosphorylation, while it dramatically enhanced Tzb-induced down-regulation of HER2 expression.

**Conclusion:**

Olive oil's bitter principle (*i.e*., oleuropein aglycone) is among the first examples of how selected nutrients from an EVOO-rich "Mediterranean diet" directly regulate HER2-driven breast cancer disease.

## Background

The growing popularity of the Mediterranean diet is due to a large body of epidemiological studies showing how the incidence of certain cancers is the lowest in the Mediterranean basin. It has been suggested that this is largely due to the relatively safe and even protective dietary habits of this area [1-9]. Olive oil is an integral ingredient of the traditional Mediterranean diet and several studies attribute many of the healthy advantages of this diet to olive oil's unique characteristics. Indeed, the relationship between the intake of olive oil and cancer risk has become a controversial issue that could have very important repercussions in human health as it may have a potential role in lowering the risk of some human neoplasms [4,7-9]. Thus, different studies have shown that the consumption of olive oil have a potential protective effect towards several malignancies, especially breast cancer (stomach, ovary, colon and endometrium cancer too). However, the final proof about the specific mechanisms by which the different components of olive oil exert their potential protective effects on the promotion and progression of human cancers requires further investigations.

Peculiar to olive oil is the abundance of the ω-9 MUFA oleic acid (OA; 18:1n-9), which ranges from 56% to 84% of total FAs, while ω-6 linoleic acid (18:2n-6), the major essential FA and the most abundant polyunsaturated FA (PUFA) in our diet, is present in concentrations between 7 and 10%. Although a single FA cannot longer be considered as an independent indicator of cancer risk, olive oil appears to unite a new composite biomarker of a low risk for breast cancer and, probably, for other types of human cancer, *i.e*., a low ω-6/ω-3 PUFA ratio and elevated ω-9 MUFA levels [10,11]. Besides this very favorable fat composition, the other fundamental characteristic of this oil is the presence of a large number of phenolic compounds including simple phenols (*e.g*., tyrosol, hydroxytyrosol, and elenolic acid), lignans (*e.g*., pinoresinol and acetoxypinoresinol) and secoiridoids (*e.g*., deacetoxy oleuropein aglycone, oleuropein aglycone and ligstroside aglycone) [12-18]. The effects of ω-9 MUFAs such as OA on cancer etiology and progression are still somewhat controversial as some epidemiological studies have revealed that intake of OA sources, particularly olive oil, appears to be protective against several carcinomas, whereas research in experimental animals has yielded inconsistent results, having reported a non-promoting or low-promoting effect to a protective one. On the other hand, little is known about the anti-cancer effects, if any, of the phenolics compounds found in olives and olive-derived products such as olive oil. Therefore, one of the remaining concerns -before going for a causal interpretation of the inverse relationship between olive oil intake and cancer risk- is to definitely establish whether olive oil-related anti-cancer effects can be explained through either the ω-9 MUFA content (*i.e*., OA) or the phenolic components of the unsaponifiable fraction.

Since cancer development and progression is believed to be a multi-step process, we recently designed a systematic approach aimed to understand the ultimate molecular mechanisms of how key cancer-related oncogenes can be regulated by olive oil's components using human cancer-derived *in vitro *models. Our findings revealed that exogenous supplementation of cultured cancer cells with physiological concentrations of the ω-9 MUFA OA drastically suppressed the expression and activity of HER2 (*erb*B-2) [9,11,19-21], one of the most commonly analyzed proto-oncogenes in human cancer studies as it plays a pivotal role in oncogenic transformation, tumorigenesis and metastasis [22-25]. Patients with HER2-overexpressing cancer are associated with unfavorable prognosis, shorter relapse time, and low survival rate [26,27]. These findings generated intense public interest, since no toxicities have been reported or suspected with OA, and suggested that supplementation with olive oil may represent a promising dietary intervention for the prevention and/or management of HER2-related carcinomas [28,29]. However, from the standpoint of molecular nutrition, a food such as olive oil cannot be considered as a drug where the active compound is concentrated and formulated in an optimal way to exert its activity. Thus, though there is no longer any doubt that olive oil has the potential to actively modulate cancer disease, it should be stressed that olive oil must be considered using a broader approach considering all the components present and not only a single one [30-34].

Here, we evaluated the anti-cancer contribution of individual olive oil's phenolic compounds [31-34]. Four representative phenolics (*i.e*., tyrosol, hydroxytyrosol, oleuropein aglycone and oleuropein glycoside) isolated from extra virgin olive oil (EVOO) -the juice of the olive obtained solely by pressing- were characterized for their abilities to affect breast cancer cell growth and survival, HER2 oncogene expression and activity and efficacy of HER2 oncogene-targeting therapies.

## Methods

### Agents

Oleuropein glycoside was obtained from Extrasynthèse (Genay, France). Tyrosol, hydroxytyrosol, oleuropein aglycone and elenolic acid were isolated from EVOO (Bertolli, Unilever Bestfoods Italia S.p.A., Inveruno-MI, Italy) by semi-preparative reverse-phase high performance liquid chromatography [Alegria Carrasco Pancorbo, Rocio Garcia Villalba, Alejandro-Vazquez Martin, Javier A. Menendez, Alberto Fernandez Gutierrez, Antonio Segura Carretero.*"*Isolation and characterization of polyphenols from polar extracts of VOO using HPLC or EC and mass spectrometry" (*Manuscript in preparation*)]. Trastuzumab (Herceptin™) was provided by Hospital Universitario de Girona Dr. Josep Trueta Pharmacy (Girona, Spain), solubilized in bacteriostatic water for injection containing 1.1% benzyl alcohol (stock solution at 21 mg/ml), stored at 4°C and used within one month. Tyrosol, hydroxytyrosol, oleuropein aglycone and elenolic acid were dissolved in 100% ethanol (stock solutions at 10 mM) and stored at -80°C until use. Oleuropein glycoside was dissolved in bacteriostatic water (stock solution at 10 mM) and stored at -80°C until use. For experimental use, EVOO phenolics and trastuzumab and were prepared freshly from stock solutions and diluted with growth medium. Control cells were cultured in medium containing the same concentration (*v/v*) as the experimental cultures with treatments. The vehicle solutions had no noticeable influence on the proliferation of experimental cells.

### Cell lines and culture conditions

MCF-7 breast cancer cells were obtained from the American Type Culture Collection (ATCC) and they were routinely grown in Dulbecco's modified Eagle's medium (DMEM, Gibco) containing 10% heat-inactivated foetal bovine serum (FBS, Bio-Whittaker), 1% L-glutamine, 1% sodium pyruvate, 50 U/ml penicillin and 50 μg/ml streptomycin. SKBR3 breast cancer cells were obtained from Dr. H. Riese (Centro Nacional de Biotecnología -CNB-, Madrid, Spain) and they were passaged in McCoy's 5 A medium (Gibco) containing 10% heat-inactivated FBS, 1% L-glutamine, 1% sodium pyruvate, 50 U/ml penicillin and 50 μg/ml streptomycin. Cells were maintained at 37°C in a humidified atmosphere of 95% air/5% CO_2_. Cells were screened periodically for *Mycoplasma *contamination.

### Construction of pBABE/HER2 retroviruses and retroviral infection

A full-length human HER2 cDNA construct in the pCMV-SPORT6 plasmid was purchased from RZPD (Berlin, Germany). The insert was excised from pCMV-SPORT6 using *Eco*RV and *Not*I sites and blunt end ligated into the pBABE-puro retroviral vector (Addgene) at the *Eco*RI site. Retroviruses were generated by co-transfection of 293T-derived phoenix cells with the retroviral constructs (pBABE, pBABE-HER2) and the packaging vector pCL-Eco by using FuGene transfection reagent (Roche Diagnostics, Barcelona, Spain) and 5 μg of each plasmid per 0.5 × 10^6 ^cells. 293T cells were cultured at 5% CO_2_, 37°C in DMEM containing 10% (*v/v*) heat-inactivated FBS. After 48 h, medium conditioned by transfected 293T cells was filtered and immediately added to MCF-7 cells. At 48 h following infection, MCF-7/HER2 cells were selected by using 2 μg/ml puromycin for 72 h. Expression of virally encoded HER2 protein was confirmed by HER2-specific ELISA analyses (see below).

### Metabolic status assessment (MTT-based cell viability assays)

The ability of EVOO phenolic compounds to affect breast cancer cell viability was determined using a standard colorimetric MTT (3–4, 5-dimethylthiazol-2-yl-2, 5-diphenyl-tetrazolium bromide) reduction assay. Cells in exponential growth were harvested by trypsinization and seeded at a concentration of ~2.5 × 10^3 ^cells/200 μl/well into 96-well plates, and allowed an overnight period for attachment. Then the medium was removed and fresh medium along with various concentrations of EVOO phenolics, trastuzumab or combinations of compounds as specified, were added to cultures in parallel. Agents were studied in combination concurrently. Control cells without agents were cultured using the same conditions with comparable media changes. Compounds were not renewed during the entire period of cell exposure. Following treatment (5 days), the medium was removed and replaced by fresh drug-free medium (100 μl/well), and MTT (5 mg/ml in PBS) was added to each well at a 1/10 volume. After incubation for 2–3 hr at 37°C, the supernatants were carefully aspirated, 100 μl of DMSO were added to each well, and the plates agitated to dissolve the crystal product. Absorbances were measured at 570 nm using a multi-well plate reader (Model Anthos Labtec 2010 1.7 reader). The cell viability effects from exposure of cells to each compound alone and their combination were analyzed as percentages of the control cell absorbances, which were obtained from control wells treated with appropriate concentrations of the compounds vehicles that were processed simultaneously. For each treatment, cell viability was evaluated as a percentage using the following equation: *(A*_570 _*of treated sample/A*_570 _*of untreated sample) × 100*.

Breast cancer cell sensitivity to EVOO phenolics and/or trastuzumab was expressed in terms of the concentration of drug required to decrease by 30%, 50% and 70% cell viability (IC_30_, IC_50 _and IC_70_, respectively). Since the percentage of control absorbance was considered to be the surviving fraction of cells, the IC_30_, IC_50 _and IC_70_values were defined as the concentration of EVOO phenolics and/or trastuzumab that produced 30%, 50% and 70% reduction in control absorbance (by interpolation), respectively. The degree of sensitization to trastuzumab by EVOO phenolics was evaluated by dividing IC_30 _values of control cells by those obtained when cells were simultaneously exposed to EVOO phenols.

### Apoptosis assays

The ability of EVOO phenolic compounds to induce apoptosis was assessed using the Cell Death Detection ELISA^PLUS ^kit obtained from Roche Diagnostics (Barcelona, Spain). Briefly, cells (5 to 10 × 10^3^/well) were grown in 96-well plates and treated, in duplicates, for 72 h with the indicated doses of hydroxytyrosol, oleuropein aglycone and oleuropein glycoside, as specified. After treatment, the 96-well plates were centrifuged (200 × *g*) for 10 min. The supernatant was discharged, lysis buffer was added, and samples were incubated at room temperature (RT) for 30 min following the manufacturer's instructions. Anti-histone biotin and anti-DNA peroxidase antibodies were added to each well and incubated at RT for 2 h. After three washes, the peroxidase substrate was added to each well, and the plates were read at 405 nm at multiple time intervals. The enrichment of histone-DNA fragments in treated cells was expressed as fold increase in absorbance as compared with control (vehicle-treated) cells.

### Conditioned medium

To prepare conditioned media, cells were plated in 100-mm tissue culture dishes and cultured in DMEM with 10% FBS until they reached 75–80% confluence. The cells were washed twice with serum-free DMEM, and incubated overnight in serum-free DMEM. Cells were then cultured for 24 h in low-serum (0.1% FBS) DMEM in the presence or absence of experimental agents, as specified. The supernatants were collected, centrifuged at 1000 *x *g, aliquoted, and stored at -80°C until testing.

### HER2- and EGFR-specific Enzyme-Linked Immunosorbent Assays

Determination of HER2 and EGFR protein content was performed with commercially available quantitative ELISAs (Oncogene Science, Bayer Diagnostics) according to the manufacturer's protocol. To assess the effects of trastuzumab and/or EVOO phenolics on HER2 and EGFR protein concentrations, breast cancer cells, after a 24 h starvation period in media without serum, were incubated with trastuzumab, oleuropein aglycone or combinations of these compounds as specified. After treatment, cells were washed twice with cold-PBS and then lysed in buffer (20 mM Tris pH 7.5, 150 mM NaCl, 1 mM EDTA, 1 mM EGTA, 1% Triton X-100, 2.5 mM sodium pyrophosphate, 1 mM β-glycerolphosphate, 1 mM Na_3_VO_4_, 1 μg/ml leupeptin, 1 mM phenylmethylsulfonylfluoride) for 30 minutes on ice. The lysates were cleared by centrifugation in an Eppendorff tube (15 minutes at 14,000 × g, 4°C). Protein content was determined against a standardized control using the Pierce Protein Assay Kit.

1:50, 1:500; 1: 5,000 and 1:10,000 dilutions of total cell lysates and conditioned medium from trastuzumab-, oleuropein aglycone- and trastuzumab + oleuropein aglycone-treated and control untreated SKBR3 and SKBR3/Tzb100 breast cancer cells were used to quantitate HER2 and EGFR protein expression in cell cultures. A standard curve was generated by using standard solutions as per manufacturer's instructions. The concentrations of HER2 and EGFR in test samples (in nanograms of HER2 and EGFR per milligram of total protein) were determined by interpolation of the sample absorbances from the standard curve. Each experiment was performed in duplicate wells.

### Activation status of HER2

Testing for phosphor-tyrosine-phosphorylation (activation) of HER2 was performed by immunoblotting procedures. Briefly, oleuropein aglycone-treated and untreated control cells were washed twice with cold PBS and then lysed as described above. Equal amounts of protein (*i.e*., 50 μg) were resuspended in 5× Laemli sample buffer for 10 min at 70°C, subjected to electrophoresis on 3–8% NuPAGE Tris-Acetate and transferred to nitrocellulose membranes. Non-specific binding on the nitrocellulose filter paper was minimized by blocking for 1 h at RT with TBS-T buffer [25 mM Tris-HCl (pH 7.5), 150 mM NaCl, 0.05% Tween 20] containing 5% (*w/v*) nonfat dry milk. The treated filters were washed in TBS-T and then incubated with a monoclonal c-*erb*B2/HER2 (phosphor-specific) antibody (Ab-18, clone PN2A; NeoMarkers, Fremont, CA, USA) for 2 h at RT in TBS-T buffer containing 1% (*w/v*) nonfat dry milk. The membranes were washed in TBS-T, horseradish peroxidase-conjugated secondary antibodies (Jackson Immunoresearch Labs) in TBS-T were added for 1 h, and immunoreactive bands were detected by chemiluminescence reagent (Pierce).

### Determination of synergism and antagonism: Isobologram analysis

The nature of the interaction between trastuzumab and oleuropein aglycone was evaluated by the isobologram technique, a dose-oriented geometric method of assessing drug interactions [35]. In the isobologram method the concentration of one agent producing a desired (*e.g*., 30% inhibitory) effect is plotted on the horizontal axis, and the concentration of another agent producing the same degree of effect is plotted on the vertical axis; a straight line joining these 2 points represents zero interaction (additivity) between two agents. The experimental isoeffect points are the concentrations (expressed relative to the IC_30 _value) of the two agents which when combined decreased cell viability by 30%. When the experimental isoeffect points fall below that line, the combination effect of the two drugs is considered to be supra-additive or synergistic, whereas antagonism occurs if the point lies above it. A quantitative index of these interactions was provided by the isobologram equation *CI*_30 _= (*a/A*) + (*b/B*), where, for this study, *A *and *B *represent the respective concentrations of trastuzumab and oleuropein aglycone required to produce a fixed level of inhibition (IC_30_) when administered alone, *a *and *b *represent the concentrations required for the same effect when trastuzumab and oleuropein aglycone were administered in simultaneous combination, and *CI*_30 _represents an index of drug interaction (Combination Index). *CI *values of < 1 indicate synergy, a value of 1 represents additivity, and values of > 1 indicate antagonism. For all estimations of CI_30_, we used only isoboles where intercept data for both axes were available.

### Statistics

Two-group comparisons were performed by the Student *t *test for paired and unpaired values. Comparisons of means of ≥ 3 groups were performed by ANOVA, and the existence of individual differences, in case of significant *F *values at ANOVA, tested by Scheffé's multiple contrasts.

## Results

### Effects of EVOO phenolic compounds on breast cancer cell viability: Oleuropein aglycone preferentially kills HER2 oncogene-overexpressing breast cancer cells

**Figure 1:**
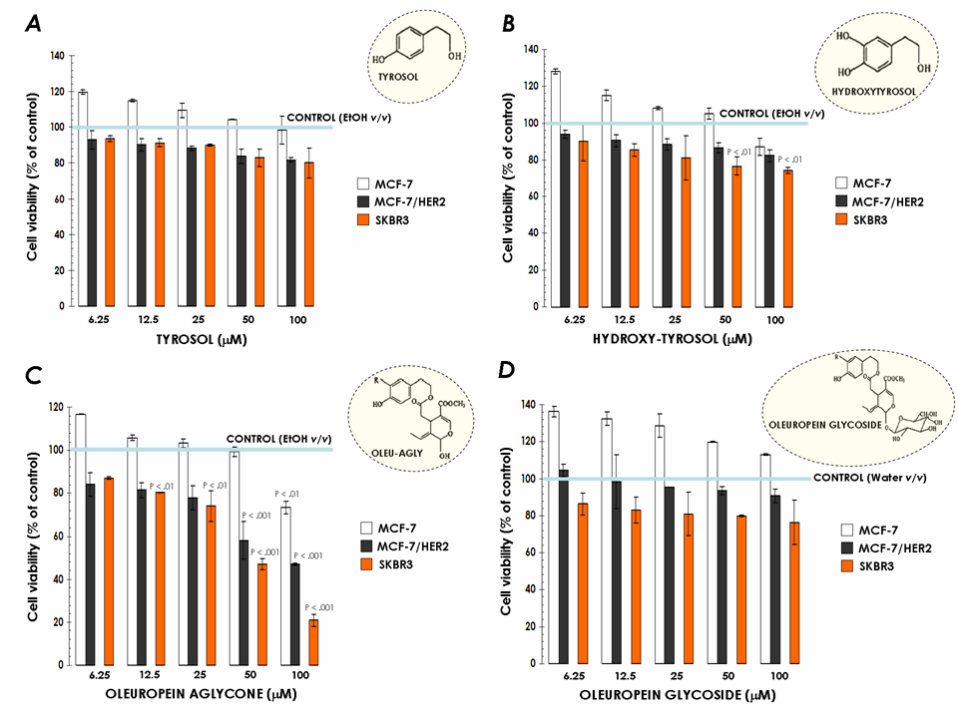
**Effects of EVOO phenolics on breast cancer cell viability**. MCF-7, MCF-7/(pBABE)HER2 and SKBR3 cells were incubated with various concentrations of tyrosol (***A***), hydroxytyrosol (***B***), oleuropein aglycone (***C***) and oleuropein glycoside (***D***) for 5 days. Cell growth, measured using MTT assay, was expressed as % of untreated cells (*blue line = 100% cell viability*). Results are means (*columns*) and 95% confidence intervals (*bars*) of three independent experiments made in triplicate. Statistically significant differences (one-factor ANOVA analysis) between experimental conditions and unsupplemented control cells are shown by asterisks (* P < .01, ** P < .001). All statistical tests were two-sided.

The anti-tumor effects of four representative EVOO phenolics were initially assessed by characterizing the metabolic status of MCF-7, MCF-7/HER2 and SK-Br3 breast cancer cell lines treated with micromolar concentrations (6.25 → 100 μM) of tyrosol, hydroxytyrosol, oleuropein aglycone, and oleuropein glycoside. MCF-7 breast cancer cells naturally express physiological levels of HER2. We stably transduced MCF-7 cells with pBABE-HER2 or pBABE (control) retroviral vectors and confirmed HER2 expression by immunoblotting (data not shown). When quantitative measurements of the concentration of HER2 cells were performed using the Human *neu *Quantitative Enzyme-Linked Immunosorbent Assay (ELISA) System (Oncogene Science, Cambridge, MA, USA) and results were expressed as nanograms of HER2 per mg of total protein, cell lysates from empty vector-transduced MCF-7/pBABE cells and MCF-7/HER2 cells were found to stably express ~100 ng HER2 mg protein^-1 ^and ~7000 ng HER2 mg protein^-1^, respectively. This level of HER2 overexpression achieved in MCF-7/(pBABE)HER2 cells (~70-fold increase when compared to MCF-7/pBABE matched control and MCF-7 parental cells) was comparable to that reported in MCF-7/Her2-18 cells, a well-characterized MCF-7-derived HER2-overexpressing clone engineered to stably express the full-length human HER2 cDNA controlled by a SV40 viral promoter [36]. SKBR3 cells represent a widely used tumor cell *in vitro *model characterized by exhibiting natural HER2 gene amplification, HER2 receptor protein overexpression (~1000 ng HER2 mg protein^-1^) and HER2-dependency for cell proliferation and survival [37]. After 5 days of treatment, cell numbers were measured using a tetrazolium salt-based (MTT) assay (Figure 1).

Treatment with tyrosol failed to reduce cell viability in the entire panel of cell lines evaluated in our study (Figure 1A). Hydroxytyrosol exposure slightly but significantly reduced cell viability at the highest concentrations tested (*i.e*., 50 and 100 μM) when used in the SKBR3 breast cancer cell line (Figure 1B). A completely different picture emerged when we evaluated the growth inhibitory effects of oleuropein aglycone: First, all the cell lines were growth-inhibited in a dose-dependent fashion upon the addition of oleuropein aglycone (Figure 1C). Second, we observed a positive correlation between the expression levels of HER2 oncogene and the efficacy of oleuropein aglycone (Table 1). Oleuropein aglycone's IC_30 _(the concentration of the compound necessary to reduce cell viability by 30% -cytostasis-) was as low as 28 μM (95% CI = 25–31 μM) in SKBR3 cells -which bear HER2 gene amplification- whereas this value was greater than 100 μM in MCF-7 cells -which express physiological levels of HER2 (*i.e*., one single copy of HER2 gene)-. Moreover, forced overexpression of HER2 in MCF-7 cells resulted in a "SKBR3-assimilated" oleuropein aglycone-sensitive phenotype. Thus, oleuropein aglycone's IC_30 _value in MCF-7/(pBABE)HER2 cells dropped to 35 μM (95% CI = 30–40 μM). This HER2-related sensitivity to oleuropein aglycone was also obvious when analyzing IC_50 _and IC_70 _cytotoxic values in MCF-7, MCF-7/(pBABE)HER2 and SKBR3 cells (Table 1). In order to investigate whether the glucose moiety in oleuropein structure affected its anti-tumoral activity, we finally evaluated the growth inhibitory effects of its precursor form (*i.e*., oleuropein glycoside; Figure 1D). Interestingly, oleuropein glycoside was ineffective at inducing any significant cytostatic or cytotoxic effect regardless of the HER2 status of breast cancer cells (Figure 1D, Table 1). These findings reveal that, among EVOO polyphenols tested, oleuropein aglycone was the most potent EVOO phenolic in decreasing breast cancer cell viability. Also of interest, it is evident that HER2 oncogene-overexpressing breast cancer cells exhibit an exacerbated sensitivity to oleuropein aglycone-induced cytotoxicity.

### Oleuropein aglycone preferentially induces apoptotic cell death in HER2-overexpressing breast cancer cells

**Figure 2:**
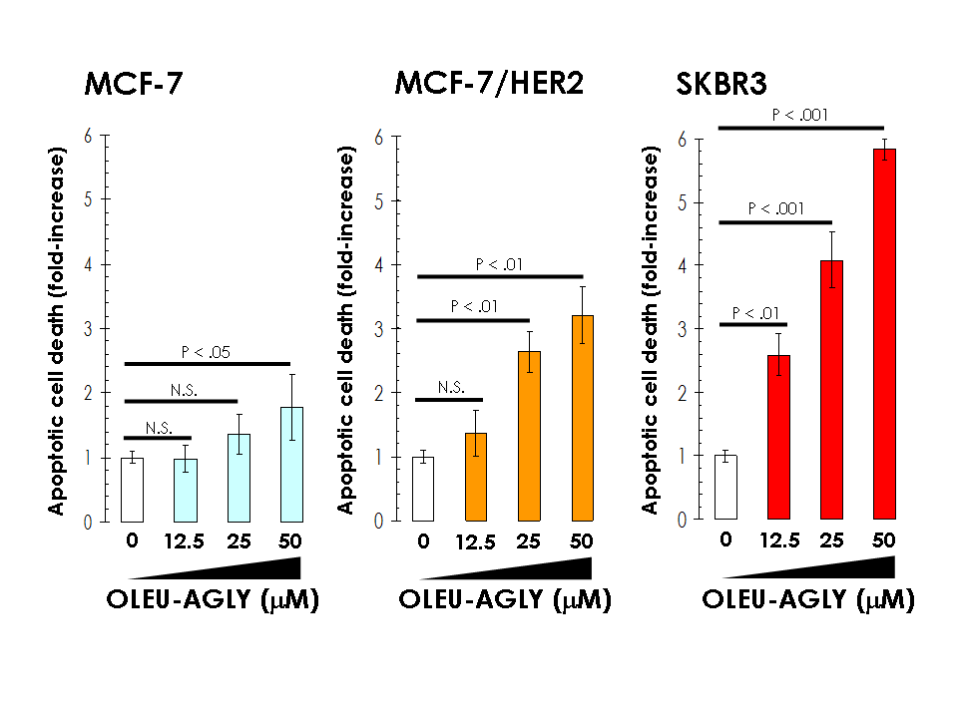
**Effects of oleuropein aglycone on breast cancer apoptotic cell death**. Quantification of apoptosis-related cell death in MCF-7, MCF-7/(pBABE)HER2 and SKBR3 cells treated with increasing concentrations of oleuropein aglycone was determined by Cell Death ELISA as described in "Material and methods". The enrichment of histone-DNA fragments in oleuropein aglycone-treated cells was expressed as fold-increase in absorbance by comparing with control (vehicle-treated) cells using the following formula: [A_405 _– A_490_]_TREATED_/[A_405_-A_490_]_UNTREATED_. Data are the mean (*columns*) and 95% confidence intervals (*bars*) of three independent experiments performed in duplicate. One-factor ANOVA was used to analyze differences in the percentage of apoptosis between the various treatment groups and the control group. * P < .01; ** P < .001; N.S.: Not statistically significant (one-factor analysis of variance). All statistical tests were two-sided.

**Figure 3:**
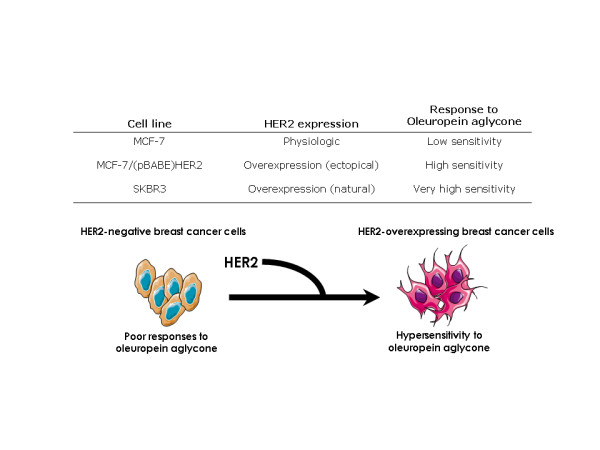
Positive correlation between HER2 overexpression and breast cancer cell sensitivity to oleuropein aglycone.

We speculated that the increased sensitivity to oleuropein aglycone that occurs in HER2-overexpressing breast cancer cells was not simply the result of changes in cell proliferation, but it might actually be due to changes in apoptotic cell death. To address this question, cells were exposed to increasing concentrations of oleuropein aglycone, apoptotic cell death was measured by a Cell Death ELISA that detects apoptosis-induced DNA-histone fragmentation, and the *x*-fold increase in apoptosis was calculated by comparing the ELISA optical density readings of treated samples, with the values of untreated cells as 1.0. HER2-negative MCF-7 cells exhibited the lowest degree of apoptosis upon treatment with oleuropein aglycone (up to 1.8-fold increase *versus *1.0-fold in untreated control MCF-7 cells; Figure 2, *left panel*). Interestingly, forced expression of HER2 in MCF-7 cells increased by ~2-fold the apoptotic effects of oleuropein aglycone. Thus, treatment with 50 μM oleuropein aglycone increased apoptotic cell death by 3.2 times in MCF-7/(pBABE)HER2 cells (Figure 2, *middle panel*). HER2-overexpressing SKBR3 cells were exquisitely sensitive to oleuropein aglycone-induced apoptosis. They exhibited the highest degree of apoptotic cell death following exposure to oleuropein aglycone (up to ~6.0-fold increase *versus *1.0-fold in untreated control SKBR3 cells; Figure 2, *right panel*). The results support the notion that HER2 overexpression associates with an increased sensitivity to oleuropein aglycone-induced breast cancer cell damage (Figure 3).

### Co-exposure to oleuropein aglycone synergistically enhances trastuzumab-efficacy in HER2-overexpressing and trastuzumab-sensitive SKBR3 breast cancer cells

We explored whether an exacerbated response of HER2-overexpressing breast cancer cells to oleuropein aglycone could be exploited to positively modulate the growth inhibitory effects of trastuzumab (Herceptin™), a humanized monoclonal antibody binding with high affinity to the ectodomain of p185^HER2 ^oncoprotein [38-40]. The sensitizing effects of oleuropein aglycone on the sensitivity of HER2-overexpressing and trastuzumab-sensitive SKBR3 cells to trastuzumab are shown in Table 2. In order to measure changes in trastuzumab efficacy in the absence or presence of oleuropein aglycone, a "sensitization factor" was determined by dividing the IC_30 _value for trastuzumab as single agent by those IC_30 _values obtained upon co-exposure to graded concentrations of oleuropein aglycone. Oleuropein aglycone enhanced the growth inhibitory effects of trastuzumab in a dose-dependent manner. Thus, as the concentration of oleuropein aglycone, the efficacy of trastuzumab was significantly augmented. Remarkably, a 50-fold increase in the anti-proliferative effects of trastuzumab was observed in the presence of 25 μg/ml oleuropein aglycone (Table 2).

**Figure 4:**
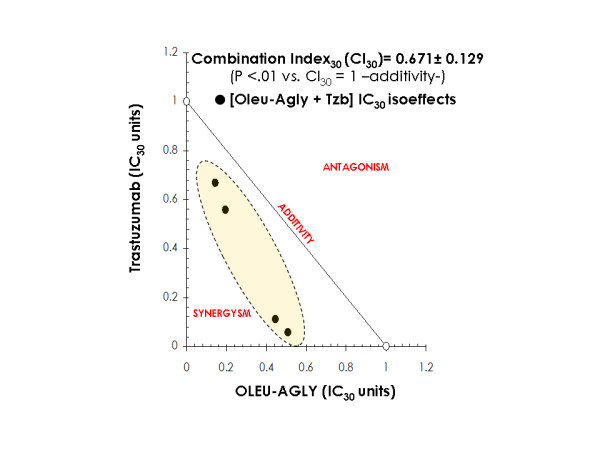
**Synergy analyses of the interaction between oleuropein aglycone and Tzb against in Tzb-sensitive SKBR3 cells**. The nature of the interaction between oleuropein aglycone and trastuzumab was evaluated by the isobologram technique, a dose-oriented geometric method of assessing drug interactions. Upon this approach, the concentration of oleuropein aglycone producing a desired (*e.g*., 30% inhibitory) effect was plotted on the horizontal axis, and the concentration of trastuzumab producing the same cytostatic degree was plotted on the vertical axis; a straight line joining these two points represents zero interaction between the two agents (CI_30 _= 1.0, additivity). An experimental isoeffect point is the concentration (expressed relative to oleuropein aglycone and trastuzumab IC_30 _concentrations) of the two agents which when combined reduced cell viability by 30%. Within the designed assay range, a set of isoeffect points was generated because there were multiple oleuropein aglycone and trastuzumab concentrations that achieved the same isoeffect. In our present study, the mean values of the survival fractions were used to generate the set of experimental isoeffect points and construct the isobole for the oleuropein aglycone-trastuzumab combination. Data points above the diagonal line of the additive effects in the isobole suggest antagonism and those below the diagonal suggest synergism. The value of the mean CI_30 _value is also labeled. Student *t *tests were applied to formally evaluate whether synergism or antagonism was evident as compared to a null-hypothesized CI_30 _of 1,0; * P < .01, ** P < .001).

Oleuropein aglycone significantly decreased cell viability of SKBR3 breast cancer cells at some of the concentrations examined. This indicated the presence of a potentially significant additive/antagonist component. Thus, possible synergistic interactions between oleuropein aglycone and trastuzumab could not be accurately discriminated from additive or antagonistic effects in the basis of the above data alone. Although there is still controversy over which method is the best for detecting true *in vitro *synergy between drug combinations, we performed a series of isobologram transformations of multiple dose-response analyses [35]. A representative transformation is presented graphically (isobologram) in Figure 4. The straight line drawn between the IC_30 _for oleuropein aglycone alone and the IC_30 _for trastuzumab alone indicates the alignment of theoretical isoeffect data points for additive interactions. The true IC_30 _values (the experimental concentrations of oleuropein aglycone and trastuzumab which combined produced 30% reduction in cell viability) were plotted and compared with the additive line. Data points above the dashed line of the additive effects in the isobole suggest antagonism, and those below the diagonal suggest synergism. In our experiments, all the experimental isoeffect data points fell in the left side of the additive line, clearly denoting a synergistic effect when combining oleuropein aglycone and trastuzumab in SKBR3 breast cancer cells. While this figure provided a graphical representation of the nature of the interaction between oleuropein aglycone and trastuzumab, the value of the mean Combination Index_30 _(CI_30_) further revealed that the amount of the two agents necessary to reduce cell viability by 30% in SKBR3 cells was only 0.671 times as much as it would be required if they demonstrated purely additive actions. The nature of the interaction obtained by using the isobologramic methodology strongly suggests that concurrent supplementation with oleuropein aglycone synergistically works to enhance the growth inhibitory activity of the anti-HER2 monoclonal antibody trastuzumab.

### Oleuropein aglycone reduces the proteolytic cleavage of the HER2 extracellular domain (ECD)

**Figure 5:**
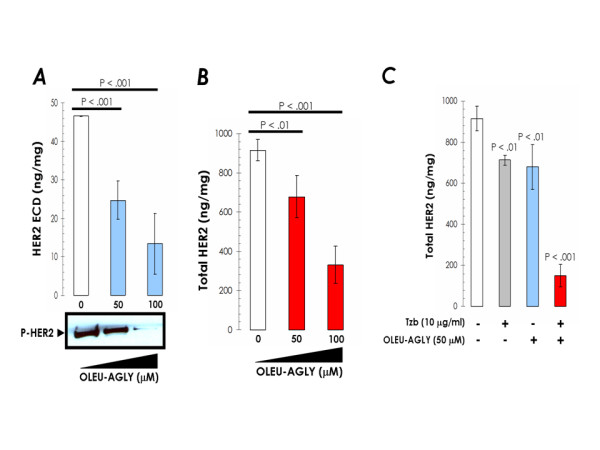
**Exogenous supplementation with oleuropein aglycone inhibits HER2 ECD cleavage and HER2 autophosphorylation (activation), down-regulates HER2 expression and synergistically enhances trastuzumab-induced depletion of HER2 in trastuzumab-sensitive SKBR3 cells**. ***A***. Overnight serum-starved SKBR3 cells were cultured in DMEM-0.1% FBS in the absence or presence of increasing concentrations of oleuropein aglycone for 24 h. ***Top***. The Oncogene Science HER2 microtiter ELISA was used according to the manufacturer's instructions to compare HER2 ECD concentrations in conditioned media from oleuropein aglycone-treated and untreated control cells. Results are means (*columns*) and 95% confidence intervals (*bars*) of three independent experiments made in triplicate. Statistically significant differences (one-factor ANOVA analysis) between experimental conditions and unsupplemented control cells are shown by asterisks (* P < .01, ** P < .001). All statistical tests were two-sided. ***Bottom***. The activation status of HER2 oncoprotein upon treatment with increasing concentrations of oleuropein aglycone was tested using a Phosphor-Tyr1248-HER2 immunoblotting procedure as described in "Material and methods". Figure shows a representative immunoblotting analysis. Equivalent results were obtained in, at least, three independent experiments. ***B, C***. Overnight serum-starved SKBR3 cells were cultured in DMEM-0.1% FBS supplemented with increasing concentrations of oleuropein aglycone in the absence or presence of trastuzumab for 72 h. The Oncogene Science HER2 microtiter ELISA was used according to the manufacturer's instructions to compare HER2 concentrations in cell pellets. Results are means (*columns*) and 95% confidence intervals (*bars*) of three independent experiments made in triplicate. Statistically significant differences (one-factor ANOVA analysis) between experimental conditions and unsupplemented control cells are shown by asterisks (* P < .01, ** P < .001). All statistical tests were two-sided.

To obtain some preliminary insights on the mechanism(s) by which oleuropein aglycone affected the efficacy of trastuzumab, we investigated the effects of oleuropein aglycone on the activation status of HER2 oncoprotein. The activation status of HER2, and not just its overexpression, is a crucial event determining both the aggressive biological behavior of HER2 positive-breast carcinomas and the responses of HER2 positive-breast tumors to chemotherapy, anti-estrogens and, more importantly for this study, to the anti-HER2 antibody trastuzumab [41-45]. Although the mechanism of activation of the HER2 oncoprotein is not completely understood, *in vitro *and *in vivo *HER2 activation has been demonstrated to occur as a consequence of proteolytic cleavage of its extracellular domain (ECD), a key event for downstream signaling [46]. Indeed, the inhibition of basal and activated cleavage of the HER2 ECD represents a key mechanism of action of trastuzumab as it prevents the production of an active (phosphorylated) form of HER2 in breast cancer cells [47]. Serum-starved SKBR3 cells were incubated for 24 h in the absence or presence of various concentrations of oleuropein aglycone (0, 50 and 100 μM). ELISA-based quantitative measurements of the concentration of HER2 ECD in the conditioned media from oleuropein aglycone-treated and untreated-control SKBR3 cells revealed that HER2 ECD significantly decreased by 47% in the presence of 50 μM oleuropein aglycone, while HER2 ECD dramatically decreased by 71% when SKBR3 cells were treated with 100 μM oleuropein aglycone (Figure 5A). To further confirm that oleuropein aglycone-induced down-regulation of HER2 ECD was indeed associated with changes in the HER2 tyrosine kinase signaling, we evaluated the phosphorylation status of the 1248 tyrosine residue (Tyr1248), which constitutes the main autophosphorylation site of HER2. When immunoblotting analyses were performed using a monoclonal c-*erb*B2/HER2 (phosphor-specific) Ab-18 (clone PN2A), which specifically recognizes the activated, tyrosine phosphorylated (P-Tyr1248) form of HER2 [48,49], we observed decreased tyrosine kinase activities (smaller PN2A bands) in oleuropein aglycone-treated SKBR3 cells (Figure 5A). These findings demonstrate that exogenous supplementation with oleuropein aglycone significantly down-regulates the concentration of HER2 ECD and, consequently, the activation status of HER2 oncoprotein.

### Oleuropein aglycone suppresses HER2 overexpression and synergistically enhances trastuzumab-induced down-regulation of HER2 oncoprotein

Considering that trastuzumab-induced inhibition of HER2 ECD cleavage precedes trastuzumab-induced down-regulation of cell surface-associated HER2 [46,47], we speculated that concurrent supplementation with oleuropein aglycone may sensitize breast cancer cells to the well-known down-regulatory actions of trastuzumab on HER2 [50,51]. To test this hypothesis, total HER2 levels were quantitatively measured by ELISA after a 72 h treatment with graded concentrations of oleuropein aglycone (0, 50 and 100 μM; Figure 5B). Total HER2 expression in cell lysates from HER2-overexpressing SKBR3 cells was reduced by 64% in the presence of 100 μM oleuropein aglycone. Moreover, the simultaneous combination of sub-optimal concentrations of trastuzumab and oleuropein aglycone reduced HER2 more than when either agent was administered alone. Thus, 10 μg/ml trastuzumab and 50 μM oleuropein aglycone induced an 84% decrease in the expression levels of HER2 when concurrently combined in SKBR3 cells, whereas when used alone, trastuzumab and oleuropein aglycone caused a 24% and 26% HER2 down-regulation, respectively (Figure 5C). These findings clearly demonstrate that the EVOO polyphenol oleuropein aglycone depletes HER2 receptor from cell-surface in breast cancer cells naturally exhibiting HER2 gene amplification and HER2 protein overexpression (*i.e*., SKBR3 cells). Moreover, a synergistic augmentation of trastuzumab-induced down-regulation of HER2 expression occurs in oleuropein aglycone-supplemented SKBR3 breast cancer cells.

### Oleuropein aglycone reverses acquired autoresistance to trastuzumab

Not all the HER2-overexpressing breast carcinomas respond to treatment with trastuzumab, and its clinical benefit is limited by the fact that resistance develops rapidly in virtually all trastuzumab-treated patients. Indeed, the majority of patients who achieve an initial response to trastuzumab develop resistance within 1 year [52-54]. This dilemma is becoming increasingly important as recent studies strongly support a role for trastuzumab in the adjuvant setting for HER2-overexpressing early-stages breast cancers [55-57]. Although it is likely that combining trastuzumab with other therapeutics for targeting HER2 should increase the magnitude and duration of response to trastuzumab [53,54], there are few data concerning novel strategies able to sensitize HER2-overexpressing breast cancer cells to the growth-inhibitory effects of trastuzumab [58]. Considering that oleuropein aglycone-promoted cell growth inhibition and apoptosis was significantly higher in HER2-overexpressing than in HER2-negative breast cancer cells, which was concomitant with a significant depletion of the HER2 oncoprotein, we hypothesized that oleuropein aglycone may represent a novel biological anti-HER2 agent capable to reverse auto-acquired resistance to trastuzumab-based treatments.

**Figure 6:**
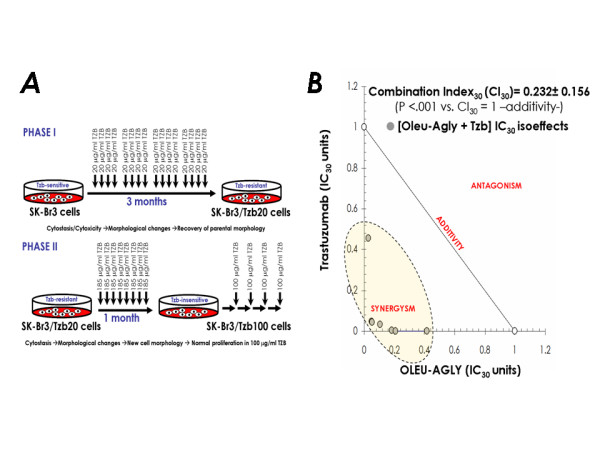
**A. Scheme for development of a new model of breast cancer autoresistance to trastuzumab**. In order to generate a preclinical model of acquired resistance to trastuzumab, HER2 gene-amplified SKBR3 cells were exposed for approximately 3 months to 20 μg/ml trastuzumab (Phase I, *top panel*). At this point, SKBR3/Tzb20 cells regained morphology similar to the parental (trastuzumab-sensitive) SKBR3 cell line. SKBR3/Tzb20 cells were then continuously grown in culture medium supplemented with 185 μg/ml trastuzumab over a period time of at least one month (Phase II, *bottom panel*). At this point, SKBR3/Tzb100 cells gained new cell morphology. Cells were then maintained in 100 μg/ml trastuzumab, a concentration of trastuzumab at which SKBR3 parental cells are strongly impaired in their metabolic status. More than 8 months later, SKBR3/Tzb100 cells normally proliferate in the presence of 100 μg/ml trastuzumab. **B. Synergy analyses of the interaction between oleuropein aglycone and Tzb against in Tzb-conditioned (high-dose resistant) SKBR3/Tzb100 cells**. The nature of the interaction between oleuropein aglycone and trastuzumab was evaluated by the isobologram technique as described in *Figure 3*.

In order to generate a preclinical model of acquired resistance to trastuzumab, HER2-overexpressing SKBR3 cells were exposed for approximately 3 months to 20 μg/ml trastuzumab. At this point and as previously reported by Nahta *et al*., cells regained morphology similar to the parental line [59]. SKBR3/Tzb20 cells were then continuously grown in culture medium supplemented with 185 μg/ml trastuzumab over a period time of at least two months. These doses were chosen because clinical trials in humans have shown plasma concentrations ranging from ~20 μg/ml to 185 μg/ml trastuzumab [58]. Cells are now maintained in 100 μg/ml trastuzumab, a concentration at which parental cells are strongly impaired in their metabolic status (Figure 6A). Indeed, the IC_30 _(30% growth inhibitory) value for trastuzumab was found to significantly increase from 18 ± 3 μg/ml in trastuzumab-sensitive SKBR3 parental cells to 110 ± 5 μg/ml in trastuzumab-conditioned (high-dose resistant) SKBR3/Tzb100 cells. Interestingly, this ~6-fold decrease in the efficacy of trastuzumab was entirely reversed when SKBR3/Tzb100 cells were co-cultured in the presence of oleuropein aglycone (Table 3). When sensitization factors were calculated by dividing the IC_30 _values of trastuzumab in the absence of oleuropein aglycone by those in the presence of oleuropein aglycone, oleuropein aglycone was found to dramatically enhance the activity of trastuzumab > 1,000 times. The precise nature of the interaction between trastuzumab and oleuropein aglycone was investigated further using the isobologram analysis (Figure 6B). When the experimental isoeffect points (the concentrations of trastuzumab and oleuropein aglycone that, when combined, produced a 30% reduction in the survival of SKBR3/Tzb100 cells) were plotted and compared with the additive line, the data points fell in the left of the line and close to the *X-Y *axes, thus suggesting a strong supra-additive or synergistic interaction between the two agents (Figure 6B). Indeed, the combined quantity of trastuzumab and oleuropein aglycone necessary to reduce SKBR3/Tzb100 cell viability by 30% was only ~0.2 times the quantity required if they demonstrated purely additive behavior (P < .001 compared with a null-hypothesized combination index of 1.0,*i.e*., additive). These findings are pioneering describing how a phytochemical naturally found in EVOO (*i.e*., oleuropein aglycone) can entirely restore trastuzumab efficacy in breast cancer cells with acquired resistance to trastuzumab.

### Oleuropein aglycone inhibits HER2 "super-expression" in trastuzumab-resistant breast cancer cells

**Figure 7:**
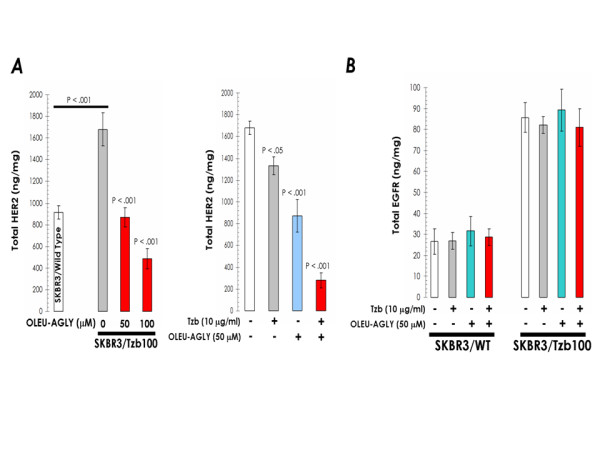
**A. Exogenous supplementation with oleuropein aglycone down-regulates HER2 expression and synergistically enhances trastuzumab-induced depletion of HER2 in trastuzumab-resistant SKBR3/Tzb100 cells**. Overnight serum-starved SKBR3/Tzb100 cells were cultured in DMEM-0.1% FBS supplemented with increasing concentrations of oleuropein aglycone in the absence or presence of trastuzumab for 72 h. The Oncogene Science HER2 microtiter ELISA was used according to the manufacturer's instructions to compare HER2 concentrations in cell pellets. **B. Oleuropein aglycone treatment does not affect EGFR (HER1) expression in trastuzumab-sensitive SKBR3 parental cell line (SKBR3/Wild Type) and in its derivative trastuzumab-resistant SKBR3/Tzb100 pool**. Overnight serum-starved SKBR3/Tzb100 cells were cultured in DMEM-0.1% FBS supplemented with increasing concentrations of oleuropein aglycone in the absence or presence of trastuzumab for 72 h. The Oncogene Science HER1 (EGFR) microtiter ELISA was used according to the manufacturer's instructions to compare HER1 concentrations in cell pellets. Results in ***A ***and ***B ***are means (*columns*) and 95% confidence intervals (*bars*) of three independent experiments made in triplicate. Statistically significant differences (one-factor ANOVA analysis) between experimental conditions and unsupplemented control cells are shown by asterisks (* P < .01, ** P < .001). All statistical tests were two-sided.

The drawbacks to our approach (*i.e*., trastuzumab-resistant pool from once cell line) are that pools may contain cells with various degrees of resistance and that the pool developed during this study may harbor resistance acquired over time or may represent a selected subpopulation of cells with inherent resistance. However, pools also represent the alterations that are represented in the majority of tumor cell populations, whereas clones -another approach that would have been used to answer our research question- represent just one isolated alteration. Nonetheless, to eliminate the possibility of HER2-independent mechanisms for the sensitizing effects of oleuropein aglycone on trastuzumab-resistant breast cancer cells, we performed HER2-specific ELISAs to quantitatively determine the expression levels of HER2 in SKBR3/Tzb100 cells. This resistant pool, when maintained in the absence of trastuzumab for 72 h, exhibited HER2 levels notably higher to those found in trastuzumab-sensitive SKBR3 parental cells (~2-fold increase; Figure 7). This "HER2 super-expression", which has not been reported in earlier trastuzumab-resistant breast cancer models [58-60], decreased back and even below the baseline HER2 level found in trastuzumab-sensitive SKBR3 parental cells when SKBR3/Tzb100 cells were treated with oleuropein aglycone (up to 71% reduction; Figure 7A). Trastuzumab exposure was still capable to reduce HER2 by 21% in SKBR3/Tzb100 cells (a percentage comparable to that found in trastuzumab-sensitive SKBR3 parental cells). Interestingly, trastuzumab treatment drastically down-regulated HER2 expression by 83% in the presence of 50 μM oleuropein aglycone, which, as single agent, decreased HER2 "super-expression" by 43% in SKBR3/Tzb100 cells.

### Oleuropein aglycone shows specificity for HER2 among other HER members

The HER2 receptor is a member of a highly homologue family of receptor tyrosine kinases (RTK) comprising c-*erb*B1 (EGFR, HER1), c-*erb*B2 (HER2), c-*erb*B3 (HER3) and c-*erb*B4 (HER4) [61-64]. Moreover, the EGFR/HER2 ratio is known to play a key role in the efficiency of the oncogenic signaling driven by HER2 overexpression [61-65]. Therefore, it could be argued that oleuropein aglycone-induced inhibition of HER2 expression may rather reflects a wider and unspecific inhibitory effect against other HER members. To test this hypothesis, we quantitatively assessed the expression levels of EGFR (HER1) following exposure to trastuzumab in the absence or presence of oleuropein aglycone. Neither trastuzumab nor oleuropein aglycone treatments alone or in concurrent combination caused detectable changes in EGFR expression in SKBR3 parental cells (Figure 7B).

Trastuzumab inhibits cell proliferation by driving cells into quiescence more effectively when EGFR content is low, while a high EGFR content renders breast cancer cells less susceptible to trastuzumab [65]. Therefore, we finally evaluated whether oleuropein aglycone altered EGFR levels in trastuzumab-conditioned SKBR3/Tzb100 cells. It is noteworthy that the acquisition of trastuzumab resistance was accompanied by up-regulatory changes in the expression levels of EGFR (~3-times when compared to those found in trastuzumab-sensitive SKBR3 parental cells). Oleuropein aglycone failed to alter EGFR expression levels in SKBR3/Tzb100 cells (Figure 7B). These data strongly suggest that, among members of the oncogenic HER network, the inhibitory effects of the EVOO polyphenol oleuropein aglycone are significantly and specifically restricted to HER2.

## Discussion

The term "Mediterranean diet" refers to dietary patterns found in olive-growing areas of the Mediterranean region and described in the 1960s and beyond. Epidemiological studies have shown that populations consuming a predominantly olive oil-based Mediterranean-style diet exhibit lower incidences of breast cancer and other chronic diseases than those eating a northern European or North American diet. Most of the attention has been so far paid to the fat components-related anti-cancer effects of the "Mediterranean diet". In this regard, we recently established the anti-oncogenic actions of the olive oil ω-9 MUFA OA in cultured cancer cells [9,11,19-21]. However, it should be noted that OA is one of the predominant FAs in largely-consumed animal foods and it is therefore unlikely that OA content is the unique responsible agent for the healthful properties of olive oil [17]. In fact, it is reasonable to hypothesize that some of the phenolic compounds present in olive oil -which have been shown to afford considerable protection against aging, coronary heart disease and cancer by inhibiting, for instance, oxidative stress [2,5,11-18]– may also exhibit "OA-like" anti-breast cancer effects. In agreement with this hypothesis, our current findings reveal that among EVOO phenolics tested, oleuropein aglycone is a potent phytochemical that significantly decreases breast cancer cell proliferation and survival by sharing OA's ability to suppress both the expression and the activity of HER2 oncogene in cultured breast cancer cells.

A significantly higher cytotoxic activity of the aglyconic, compared with the glycosidic, form of oleuropein was clearly observed in all the breast cancer cell lines employed in our study. This difference possibly derives from the greater lipophilicity of the former, a property that should allow better cell membrane incorporation and/or interaction with other lipids [66,67]. Since an important step in the body metabolism of EVOO polyphenols might be that oleuropein aglycone is split into hydroxytyrosol or tyrosol and elenolic acid [13,68-70], it will be relevant to evaluate whether the dihydroxy moiety of oleuropein (*i.e*., hydroxytyrosol) and/or the iridoid moiety (*i.e*., elenolic acid) can recapitulate (entirely or solely in part) oleuropein aglycone-induced breast cancer cell growth inhibition and apoptotic breast cancer cell death. Our findings strongly suggest that: First, upon β-glucosidase-induced deglucosidation of the iridoid glycoside moiety of oleuropein (*i.e*., activation of the glycoside form of oleuropein) [71], the aglyconic form of oleuropein can exhibit growth inhibitory and pro-apoptotic activities against human breast cancer cells. Second, although the hydroxytyrosol moiety was notably less cytotoxic than oleuropein aglycone it was still capable to significantly promote breast cancer apoptotic cell death. However, the elenolic acid moiety notably loses the anti-breast cancer actions of oleuropein aglycone (data not shown). Although the ultimate reason determining the pro-apoptotic potencies of EVOO phenolics should be analyzed in future studies, it is reasonable to argue that EVOO phenolic compounds with a simple structure (*i.e*., involving only a single phenol ring) cannot exert anti-breast cancer actions, and that a more complex structure (*i.e*., involving two or more phenol rings) may be required to exert cytotoxic effects in breast cancer cells. In accordance with this idea, Carluccio *et al*. reported that oleuropein aglycone was much more potent than tyrosol or hydroxytyrosol in decreasing adhesion molecule expression on cultured human endothelial cells [72]. Also, the anti-oxidant properties of oleuropein aglycone's moieties, which are similarly exhibited by hydroxytyrosol but they disappear in the case of elenolic acid [16-18,66,67,72], may account for the dissimilar ability of EVOO phenolics to inhibit breast cancer cell growth.

**Figure 8:**
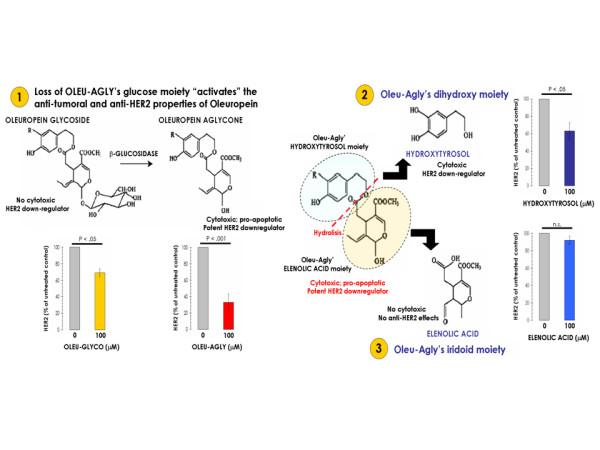
**Relationship between oleuropein metabolism, apoptosis, and HER2 expression in SKBR3 breast cancer cells**. In order to evaluate how oleuropein aglycone's metabolism affects breast cancer cell viability, breast cancer apoptotic cell death, and HER2 oncogene expression, we performed MTT-based metabolic assays (as described in *Figure 1*), Cell Death ELISAs (as described in *Figure 2*), and HER2-specific ELISAs (as described in *Figure 5*) in serum-starved SKBR3 cells cultured in the absence or presence of equimolar concentrations of oleuropein glycoside (*i.e*., the precursor form of oleuropein aglycone), oleuropein aglycone, hydroxytyrosol (*i.e*., the dihydroxy moiety of oleuropein aglycone), and elenolic acid (*i.e*., the iridoid moiety of oleuropein aglycone). Results for HER2 protein expression are means (*columns*) and 95% confidence intervals (*bars*) of three independent experiments made in triplicate. Statistically significant differences (one-factor ANOVA analysis) between experimental conditions and unsupplemented control cells are shown by asterisks (* P < .01, ** P < .001). All statistical tests were two-sided. Figure shows both the chemical structures and the metabolic pathway of the EVOO phenolic compounds studied.

Oleuropein has recently been shown to efficiently inhibit the growth of T47D breast cancer cells, and its growth inhibitory effect was mediated, at least in part, by acting as cytoskeleton disruptor [73]. T47D cells express physiological levels of the HER2 oncogene. Here, we reveal that inhibition of HER2 expression/activity is a previously unrecognized molecular mechanism through which the EVOO polyphenol oleuropein aglycone can exert its tumoricidal effects in human breast cancer cells. This anti-HER2 action, in turn, synergistically works with the anti-HER2 monoclonal antibody trastuzumab to down-regulate HER2 expression. Moreover, upon development of trastuzumab-resistant breast cancer cells, oleuropein aglycone was found to fully overcome trastuzumab resistance while suppressing the extremely high levels of HER2 occurring in trastuzumab-conditioned (high-dose resistant) SKBR3/Tzb100 cells. Although the ultimate molecular mechanism underlying oleuropein aglycone-induced down-regulation of HER2 remains to be elucidated, our current findings, altogether, strongly suggest that: *a*.) it should be, at least in part, different to that of trastuzumab (*i.e*., targeting of HER2 ECD and subsequent HER2 receptor degradation) as SKBR3/Tzb100 cells did not exhibit cross-resistance to the anti-HER2 effects of oleuropein aglycone; and *b*.) it should be, at least in part, similar to that underlying trastuzumab-resistance and "HER2 super-expression" because co-treatment with oleuropein aglycone restored the efficacy of trastuzumab while blocking up-regulation of HER2 in SKBR3/Tzb100 cells. It has been reported that oleuropein and β-glucosidase have been developed as a set to provide a chemical defense against herbivores or pathogens [71]. Thus, after activation, oleuropein aglycone has strong protein-denaturing/protein-crosslinking/lysine-decreasing activities, which appear to be caused by both the glutaraldehyde-like structure formed after deglucosidation of the iridoid glycoside moiety and the quinone structure formed after oxidation of the dihydroxy moiety of oleuropein [71]. When we evaluated oleuropein aglycone's metabolism-related anti-tumoral activity in SKBR3 breast cancer cells, we observed that oleuropein deglucosidation not only enables its pro-apoptotic effects but further enhances its anti-HER2 effects (Figure 8, *left panel*). As suggested above, these findings clearly establish that the interaction with the cell membrane is a key step regulating the anti-breast cancer effects of oleuropein aglycone. Moreover, the main metabolites of oleuropein aglycone (*i.e*., hydroxytyrosol and elenolic acid) are not molecularly equivalent in their ability to diminish HER2 expression (Figure 8, *right panel*). Therefore, it is reasonable to suggest that oleuropein aglycone may function, at least in part, through proteasomal degradation of members of the HER family induced by the quinone structure formed after oxidation of its dihydroxy moiety, as recently described for other oleuropein-closely related polyphenols such as apigenin [74]. On the other hand, treatment with oleuropein aglycone failed to modulate the expression levels of HER2-closely related HER receptors such as HER1 (EGFR), thus suggesting that oleuropein aglycone may indirectly suppress HER2 overexpression at the transcriptional level by suppressing HER2 promoter activity. We are currently evaluating whether the inhibitory effects of oleuropein aglycone on HER2 expression are dependent on the molecular mechanism eliciting HER2 protein overexpression in breast cancer cells.

Caution must be applied when extrapolating *in vitro *results into clinical practice. Regarding the safety of oleuropein in humans, in acute toxicity studies no lethality or adverse effects were observed in mice even when it was administered at doses as high as 1000 mg/kg [73,75]. Moreover, several human studies have been conducted with olive extracts or its polyphenolics showing no adverse effects [76-78]. Another important issue is whether food-derived polyphenols are absorbed and retain their properties *in vivo*. A Mediterranean diet rich in EVOO can supplies ~10–20 mg of polyphenols per day and, although the bioavailability of different phenolic compounds varies, it appears that those from EVOO are well absorbed in humans [69,79]. Also, one limitation of our current results is that the phenolics that were active (*i.e*., oleuropein aglycone) had anti-breast cancer effects at concentrations that are unlikely to be achieved *in vivo*. Considering that plasma concentration of these compounds will exceed 10^-6^-10^-5 ^M [69,79,80], the tumoricidal and anti-HER2 oncogene effects at micromolar concentrations of oleuropein aglycone are within the concentration range expected after nutritional intake from EVOO-rich Mediterranean diets.

## Conclusion

**Figure 9:**
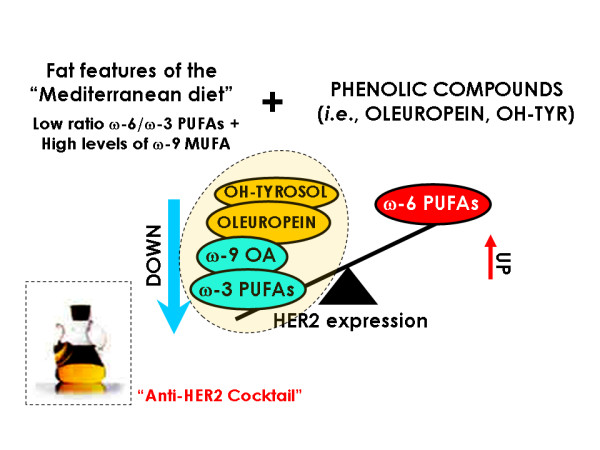
**Olive oil as a naturally-occurring "anti-HER2 cocktail"**. In terms of protective effects against breast cancer, olive oil exhibits a very favorable fat composition as it appears to unite a new composite biomarker for breast cancer,*i.e*., a low ω-6/ω-3 PUFA ratio and elevated ω-9 MUFA levels. We previously demonstrated that ω-3 PUFAs such as alpha-linolenic acid (ALA) and docosahexaenoic acid (DHA) as well as the ω-9 MUFA oleic acid (OA) significantly inhibit HER2 expression and HER2-driven oncogenic activity in breast cancer cells naturally exhibiting HER2 gene amplification and HER2 protein overexpression [9, 11, 19-21, 81, 82]. Conversely, the ω-6 PUFA linoleic acid (LA) was found to significantly activate and up-regulate HER2. Since HER2 plays a pivotal role in malignant transformation, tumorigenesis, metastasis, and treatment failure in breast cancer disease, our current findings showing that blockade of HER2 expression and HER2-driven oncogenic activity also represents a new molecular mechanism of quantitatively minor phenolic components of olive oil such as oleuropein and hydroxytyrosol (OH-Tyr) strongly support the notion that olive oil may be extremely efficient at inhibiting HER2-regulated breast cancer etiology and metastatic progression.

In summary, our findings demonstrate for the first time a new molecular mechanism by which quantitatively minor phenolic components of EVOO such as oleuropein aglycone, through the specific inhibition of HER2 oncogene, may exert protective effects not only in the promotion (risk) but further in the progression (invasion and metastasis) of human breast cancer. Together with EVOO ω-9 MUFA OA, the bitter principle of olives and olive oil oleuropein aglycone is among the first examples of how selected nutrients from the so-called "Mediterranean Diet" directly regulate the expression and activity of HER2, a *proto*-oncogene that plays a pivotal role in malignant transformation, tumorigenesis, metastasis, and treatment failure in breast cancer disease (Figure 9). The ability of oleuropein aglycone to exhibit synergistic antitumor effects when concurrently given to breast cancer cells chronically exposed to trastuzumab for several months further underscores the clinical relevance of these findings as they reveal a novel approach capable to circumvent trastuzumab resistance in breast cancer disease.

## Abbreviations

EVOO: Extra Virgin Olive Oil

HPLC: High Pressure Liquid Chromatography

Tzb: Trastuzumab

ELISA: Enzyme-Linked Immunosorbent Assays

CI: Combination Index

ECD: Extracellular Domain

IC: Inhibitory Concentration

FA: Fatty Acid

MUFA: Monounsaturated Fatty Acid

## Competing interests

The author(s) declare that they have no competing interests.

## Authors' contributions

JAM and AVM performed cell viability, apoptosis, ELISAs and drug treatments. JAM, AVM and ASC were responsible for data analysis. ACP and RGV performed semi-preparative reverse-phase HPLC for isolation of EVOO polyphenols and coordinated the chemical (ACP, RGV, AGF, ASC) and biological (JAM, AVM, RC) study groups. RC, JB and AFG participated in the design and coordination of the study design. JAM and ASC conceived the study, participated in its design, coordination and in the draft of the manuscript. All authors read and approved the final version of the manuscript.

## Pre-publication history

The pre-publication history for this paper can be accessed here:



## Figures and Tables

**Table 1:** Effects of EVOO phenolics on breast cancer cell viability

	**TYROSOL**			**HYDROXYTYROSOL**			**OLEUROPEIN AGLYCONE**			**OLEUROPEIN GLYCOSIDE**		
	IC_30_	IC_50_	IC_70_	IC_30_	IC_50_	IC_70_	IC_30_	IC_50_	IC_70_	IC_30_	IC_50_	IC_70_
**MCF-7**	N.A.	N.A.	N.A.	N.A.	N.A.	N.A.	~**105**	N.A.	N.A.	N.A.	N.A.	N.A.
**MCF-7/HER2**	N.A.	N.A.	N.A.	N.A.	N.A.	N.A.	**35 (95% CI = 30–40)**	**85 (95% CI = 82–88)**	N.A.	N.A.	N.A.	N.A.
**SK-Br3**	~**120**	N.A.	N.A.	~**110**	N.A.	N.A.	**28 (95% CI = 25–31)**	**47 (95% CI = 43–51)**	**82 (95% CI = 80–84)**	~**110**	N.A.	N.A.

The metabolic status of tyrosol-, hydroxytyrosol-, oleuropein aglycone-, and oleuropein glycoside-treated MCF-7, MCF-7/(pBABE)HER2 and SKBR3 breast cancer cells was evaluated using a MTT-based cell viability assay and constructing dose-response curves as described in "Methods". Inhibitory Concentrations (IC) producing the IC_30_, IC_50 _and IC^70 ^values (the concentration of each agent needed to reduce cell viability by 30%, 50% and 70% relative to untreated control cells, respectively) were calculated by interpolation. Values are means (in μM) and 95% confidence intervals (95% CI) of three independent experiments made in triplicate.

N.A. Not Available.

**Table 2:** Effects of oleuropein aglycone on the sensitivity of SKBR3 cells to trastuzumab ^a^

**Oleuropein aglycone (μM)**	**TRASTUZUMAB IC**_**30**_** (μg/ml)**	**Sensitization factor (*x*-fold)**^**b**^
**0**	**18**	**-**
3.125	10	**2**
6.25	4.5	**4**
12.5	1.1	**16**
25	0.36	**50**

^a ^Data represent the cell viability effects upon simultaneous exposure of HER2-positive and trastuzumab-sensitive SKBR3 breast cancer cells to oleuropein aglycone and trastuzumab. Briefly, SKBR3 cells were incubated in serial dilutions of trastuzumab in the absence or presence of a given concentration of oleuropein aglycone. IC_30 _values are the concentrations of trastuzumab which decreased cell viability by 30%, measured by using a MTT assay as described in "Methods". Values are mean of three independent experiments carried out in triplicate.

^b ^Sensitization factors were obtained by dividing the IC_30 _value of trastuzumab as single agent by the IC_30 _values obtained when oleuropein aglycone was concomitantly supplemented.

**Table 3:** Effects of oleuropein aglycone on the sensitivity of SKBR3/Tzb100 cells to trastuzumab ^a^

**Oleuropein aglycone (μM)**	**TRASTUZUMAB IC**_**30**_** (μg/ml)**	**Sensitization factor (*x*-fold)**^**b**^
**0**	**110**	**-**
3.125	3.7	**30**
6.25	0.13	**846**
12.5	0.045	**> 1,000**
25	0.0075	**> 1,000**

^a ^Data represent the cell viability effects upon simultaneous exposure of HER2-positive and trastuzumab-conditioned (resistant) SKBR3/Tzb100 breast cancer cells to oleuropein aglycone and trastuzumab. Briefly, SKBR3 cells were incubated in serial dilutions of trastuzumab in the absence or presence of a given concentration of oleuropein aglycone. IC_30 _values are the concentrations of trastuzumab which decreased cell viability by 30%, measured by using a MTT assay as described in "Methods". Values are mean of three independent experiments carried out in triplicate.

^b ^Sensitization factors were obtained by dividing the IC_30 _value of trastuzumab as single agent by the IC_30 _values obtained when oleuropein aglycone was concomitantly supplemented.
